# Supplementary material for: Deletion of wfs1 Impairs Oligodendrocyte Precursor Cells Dorsal Distribution and Myelination Through the wfs1-hmgcs1 Axis in Zebrafish
Source: Biology (Basel). 2026 Aug 16;15(16):1402. doi: 10.3390/biology15161402 (PMC13510151; doi:10.3390/biology15161402)
Supplement: Supplementary file 1 [file biology-15-01402-s001.zip › File S1-Transcriptome sequencing data and differentially expressed genes (DEGs) in wfs1-- zebrafish at 5 dpf/1.GO/wfs1KOvsWT/DOWN/wfs1KOvsWT.down_GObar.pdf]

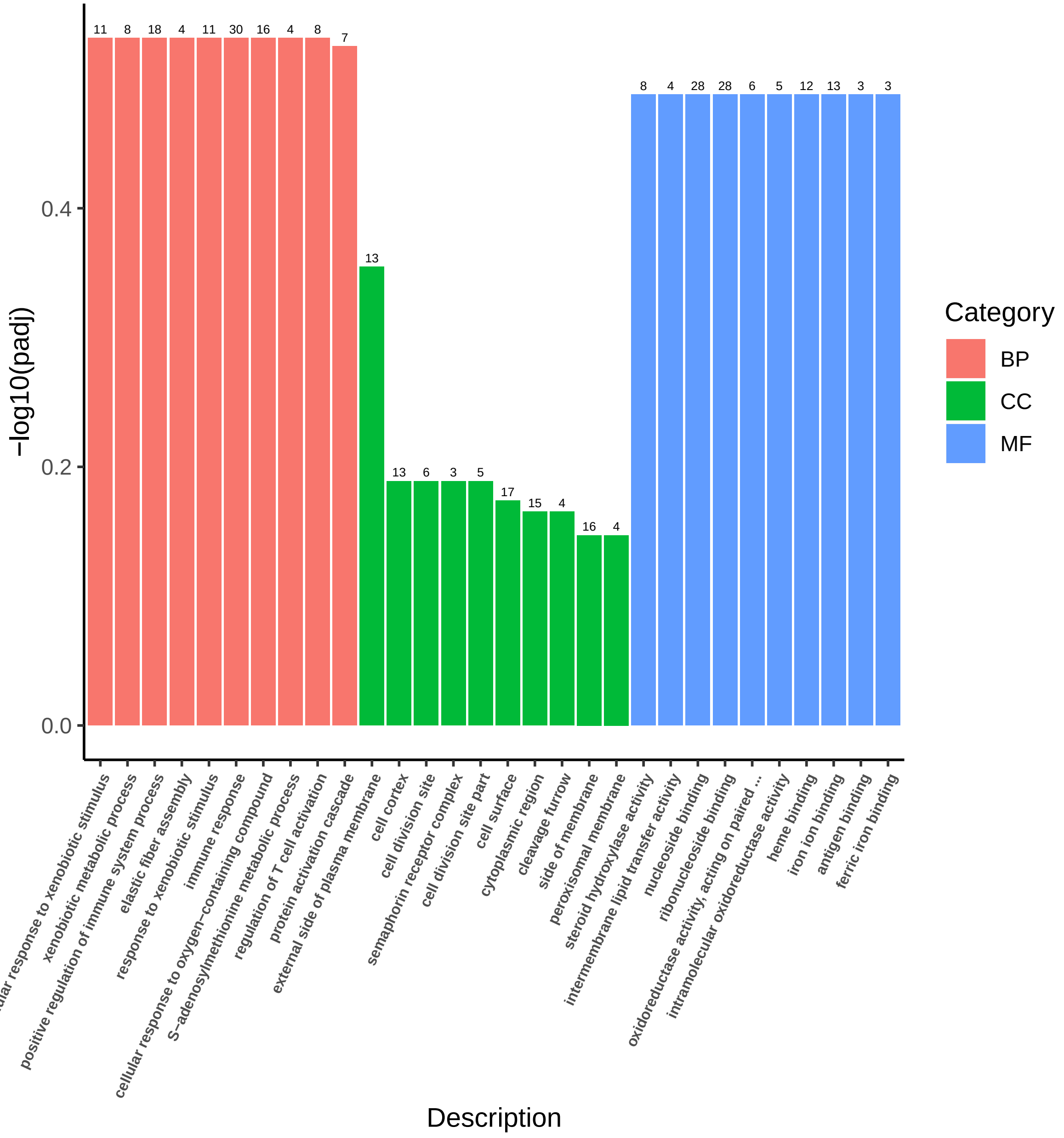

−log10(padj)
0.0
0.2
0.4
Description
Category
BP
CC
MF
11
8
18
4
11
30
16
4
8
7
13
13
6
3
5
17
15
4
16
4
8
4
28
28
6
5
12
13
3
3
cellular response to xenobiotic stimulus
xenobiotic metabolic process
positive regulation of immune system process
elastic fiber assembly
response to xenobiotic stimulus
immune response
cellular response to oxygen−containing compound
S−adenosylmethionine metabolic process
regulation of T cell activation
protein activation cascade
external side of plasma membrane
cell cortex
cell division site
semaphorin receptor complex
cell division site part
cell surface
cytoplasmic region
cleavage furrow
side of membrane
peroxisomal membrane
steroid hydroxylase activity
intermembrane lipid transfer activity
nucleoside binding
ribonucleoside binding
oxidoreductase activity, acting on paired ...
intramolecular oxidoreductase activity
heme binding
iron ion binding
antigen binding
ferric iron binding
